# *Shigella* Infection Interferes with SUMOylation and Increases PML-NB Number

**DOI:** 10.1371/journal.pone.0122585

**Published:** 2015-04-07

**Authors:** Saima M. Sidik, Jayme Salsman, Graham Dellaire, John R. Rohde

**Affiliations:** 1 Department of Microbiology and Immunology, Dalhousie University, Halifax, NS, Canada; 2 Department of Pathology, Dalhousie University, Halifax, NS, Canada; 3 Departments of Pathology and Biochemistry & Molecular Biology, Dalhousie University Halifax, NS, Canada; 4 Beatrice Hunter Cancer Research Institute, Halifax, NS, Canada; University of Regensburg, GERMANY

## Abstract

Shigellosis is a severe diarrheal disease that affects hundreds of thousands of individuals resulting in significant morbidity and mortality worldwide. Shigellosis is caused by *Shigella* spp., a gram-negative bacterium that uses a Type 3 Secretion System (T3SS) to deliver effector proteins into the cytosol of infected human cells. *Shigella* infection triggers multiple signaling programs that result in a robust host transcriptional response that includes the induction of multiple proinflammatory cytokines. PML nuclear bodies (PML-NBs) are dynamic subnuclear structures that coordinate immune signaling programs and have a demonstrated role in controlling viral infection. We show that PML-NB number increases upon *Shigella* infection. We examined the effects of *Shigella* infection on SUMOylation and found that upon *Shigella* infection the localization of SUMOylated proteins is altered and the level of SUMOylated proteins decreases. Although *Shigella* infection does not alter the abundance of SUMO activating enzymes SAE1 or SAE2, it dramatically decreases the level of the SUMO conjugating enzyme Ubc9. All *Shigella*-induced alterations to the SUMOylation system are dependent upon a T3SS. Thus, we demonstrate that *Shigella* uses one or more T3SS effectors to influence both PML-NB number and the SUMOylation machinery in human cells.

## Introduction

*Shigella* spp. (hereafter refered to as *Shigella*) is the causative pathogen of shigellosis: a severe diarrheal disease that inflicts a major disease burden on the developing world. *Shigella* damages the colonic epithelium, resulting in massive inflammation and a characteristic bloody diarrhea. *Shigella* are gram negative bacteria that use a type three secretion system (T3SS) to inject effector proteins into the cytosol of infected human cells (reviewed in [1]). Most virulence factors, including the components of the T3SS, are encoded by a large (220 kb) plasmid that is essential for virulence. The repertoire of effectors for the *Shigella* T3SS includes many factors that interfere with normal signal transduction in host cells.

T3SS effector proteins are adept at mimicking eukaryotic protein function, even in cases where the signaling components may be completely lacking in bacterial systems [2]. For example, despite the ubiquitin system being absent in prokaryotes *Shigella* possess many enzymes that act on the ubiquitin pathway. The ubiquitin system is a post-translational modification pathway that controls many eukaryotic processes through alteration of protein stability, localization, and/or activation of enzymatic function [3]. Of the approximately 50 predicted *Shigella* effectors, at least 14 are dedicated to interfering with or utilizing the ubiquitin system (reviewed in [2, 4]). *Shigella* effectors that target the Ub system include a class of E3 ubiqutin ligases, the IpaH family [5]. *Shigella* also use OspG, a protein kinase, that interacts with charged ubiquitin conjugating enzymes to downregulate inflammation [6, 7]. Finally, *Shigella* also possess OspI; a deamidase that inactivates Ubc13, which is responsible for formation of K63-linked ubiquitin chains, resulting in reduced signaling through the NF-κB cascade[8]. The ability of *Shigella* to alter ubiquitin-dependent pathways in the host cell is one example of how this intracellular pathogen subverts its host during successful infection. Given that ubiquitin-like modifications often work in concert with the ubiquitin pathway, it is possible that *Shigella* may possess mechanisms to interfere with these programs [9, 10].

The small ubiquitin-like modifier (SUMO) system is also absent in prokaryotes and is a regulator of diverse processes including genome stability, intracellular transport, immune responses, and transcription [11–13]. Like ubiquitin, SUMOylation is a reversible protein modification where SUMO, a 10kDa protein, is covalently attached to lysine residues of target proteins[14]. SUMOylation, like the ubiquitin system, involves the sequential transfer of SUMO from E1 enzymes (encoded by SAE1 and SAE2) to an E2 conjugating enzyme [15]. In contrast to the ubiquitin system, which may utilize dozens of E2 enzymes, the SUMO system relies on a single enzyme, Ubc9. In addition, there are three isoforms of SUMO (SUMO1, SUMO2 and SUMO3) expressed in human cells, and target proteins may be modified by one or a combination of these isoforms. The impact of *Shigella* infection on the SUMO pathways is currently unknown; however, it has been shown that *Listeria monocytogenes* (hereafter *Listeria*), a bacterium that shares many similarities with *Shigella* in infection strategies, impairs the SUMO pathway[16]. *Listeria* use a secreted pore-forming toxin LLO to impair SUMOylation of eukaryotic proteins and gain an advantage in intracellular replication. *Listeria* infection results in a global reduction in eukaryotic SUMOylation by destabilizing Ubc9 [16]. Similar strategies have been described for many viruses as well (reviewed in [12]). Very recently, it was shown that a virulence factor for the obligate intracellular pathogen, *Ehrlichia chaffeensis*, is SUMOylated [17]. In that study, it was shown that SUMO1-conjugated proteins colocalized with bacterial inclusions and that impairing SUMOylation resulted in a decreased ability for *Ehrlichia chaffeensis* to replicate. Another recent report similarly demonstrated that AmpA, a secreted virulence factor for *Anaplasma phagocytophilum* was also SUMOylated and that pharmacological inhibition of the SUMO program resulted in a significant decrease in bacterial load of infected cells [18]. Thus, while the role of SUMOylation in immunity is emerging, it is clear that successful pathogens have evolved mechanisms to alter or to hijack SUMOylation.

Mammalian cells sense the presence of pathogens using Pattern Recognition Molecules (PRMs). Engagement of PRMs with their microbial ligands, such as peptidoglycan components or flagellin, result in the activation of signaling cascades that activate gene expression (reviewed in [19]). Successful pathogens produce factors that interfere with immune signaling cascades that govern transcription responses, and in some cases can interfere directly with transcription machinery [20]. Within the nucleus, many immune responsive signaling programs converge on subnuclear structures known as promyelocytic leukemia nuclear bodies (PML-NBs) [21, 22]. PML-NBs are dynamic macromolecular structures that coordinate a variety of important cellular processes including anti-viral activity, DNA damage responses, gene expression and cell fate decisions such as apoptosis and senescence [22, 23]. Although the PML protein is an absolute requirement for the formation of PML-NBs, these bodies are structurally heterogeneous with over 100 different cellular proteins associating with PML-NBs either constitutively (e.g. SP100, DAXX) or transiently in response to various stimuli or stresses (e.g. p53) [24–26]. SUMOylation is important for PML-NB formation and function as many proteins that associate with PML-NBs are either SUMO-modified and/or contain SUMO-interacting motifs, including PML itself [27].

In addition to an association with tumour suppression and DNA repair [28], several lines of evidence support a role for PML-NBs in the immune response to pathogens. First, PML-NBs form part of an intrinsic antiviral response through their ability to silence gene expression from certain DNA virus genomes, such as members of the *Herpesviridae* family, upon infection [12, 29, 30]. Second, PML-NBs are key players in orchestrating the interferon (IFN) response that is triggered by viral and some other intracellular infections and the number of PML-NBs increases upon IFN treatment [31]. Third, viruses have developed specific countermeasures that disrupt PML organization and function [32–36]. To date, the role of PML-NBs in bacterial infection has not been explored, however PML is required for sensitivity to lippopolysaccharide (LPS)-induced septic shock indicating a role for PML in innate immunity to bacterial pathogens [37].

The SUMOylation program was recently shown to play a protective role against *Shigella* infection [38]. Quantitative proteomic studies showed that *Shigella* infection results in the SUMO-2 modification of many transcriptional regulators that coordinate the inflammatory response. Overexpression of components of the SUMO pathway significantly decreased the ability of *Shigella* to enter cultured epithelial cells. These *in vitro* results were consistent with results obtained in an infection model of haploinsufficient *UBC9* ^+/-^ mice. When compared with wild type controls, newborn *UBC9* ^+/-^ mice Infected with *Shigella* displayed a dramatic increase in the destruction of the colonic tissue, an increase in gut permeability, and an increase in proinflammatory cytokines. Taken together, these data show that the SUMO pathway restricts *Shigella* infection at multiple steps [38].

Given the importance of the SUMO and PML pathways in innate immunity to multiple cellular pathogens, we assessed the impact of *Shigella* infection on both PML-NBs and SUMOylation. Our findings indicate that PML-NB number and SUMOylation of cellular proteins are altered during infection with *Shigella*, and these cellular changes require T3SS effectors.

## Results

### *Shigella* infection increases the number of PML bodies in infected cells

**Fig 1:**
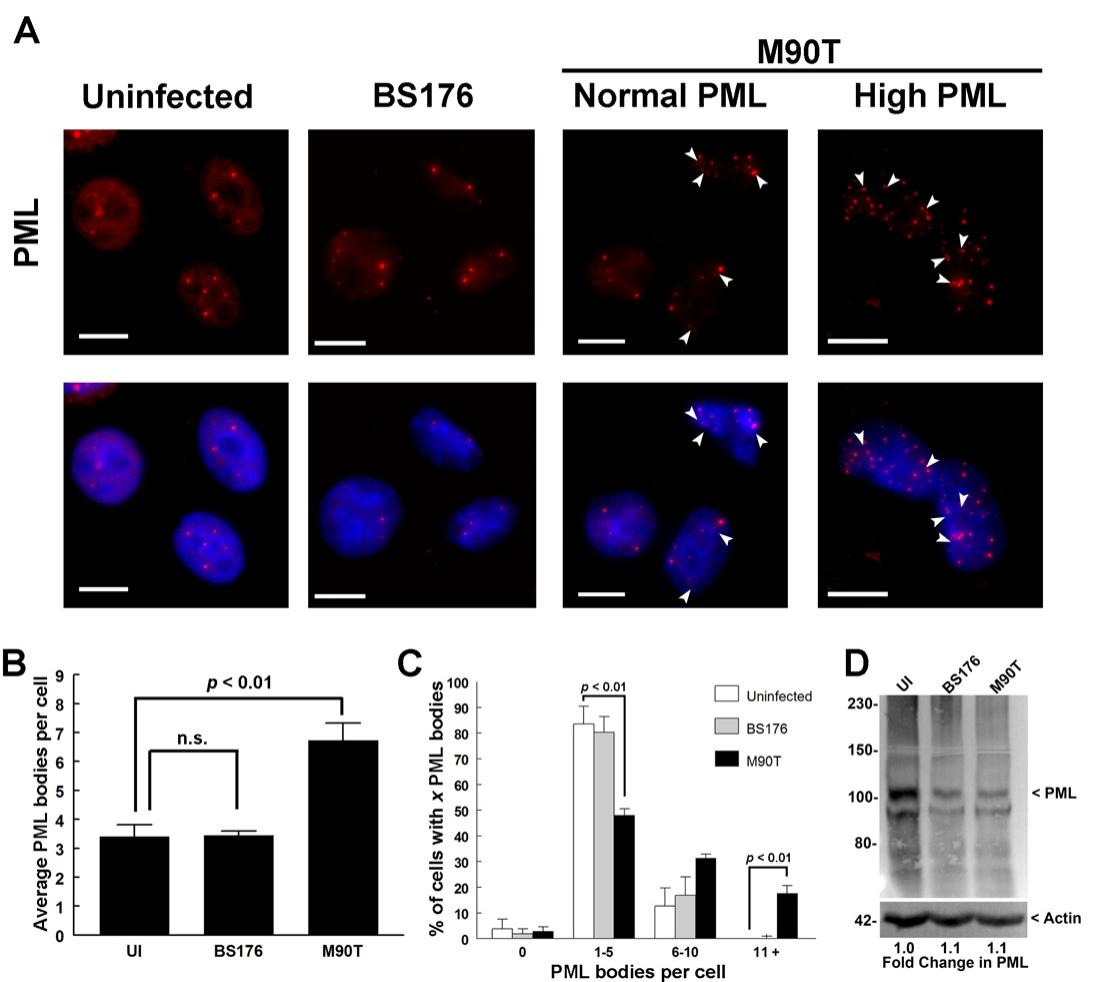
*Shigella* infection increases PML-NB number. HeLa cells were not infected (UI), infected with a non-invasive strain (BS176), or infected with an invasive wild-type strain (M90T) as indicated, both strains contained a plasmid expressing the *afaE* adhesin (A). Cells were incubated for 3 hours, fixed and immunostained for PML (red). M90T-infected cells with normal and high levels of PML nuclear bodies are shown as indicated. DNA was visualized with DAPI (blue) and white arrow heads indicate PML NB “doublets”. Scale bars = 10μm. The average number of PML bodies per cell for each treatment condition was quantified (B) and the *p*-value of Student’s t-test was calculated (n.s. = not significant). The percentage of cells containing either 0, 1–5, 6–10 or 11+ PML bodies was also determined (C) and the *p*-value of Student’s t-test was calculated. Values in B and C represent the mean +/- SE, n = 3. Total PML protein levels for each treatment condition was determined by Western blot analysis (D).

PML-NBs are heterogeneous complexes of nuclear factors that have been implicated in genome integrity, immune function, and stress [39]. We examined PML-NB dynamics in HeLa cells infected with *Shigella* that contained a plasmid expressing the afimbrial adhesin *afaE*. Infection with wild-type *Shigella* (M90T) caused a large increase in PML NBs in many of the infected cells (Fig 1A). We quantified the number of PML-NBs per cell and found that uninfected cells had an average of 3.4 PML-NBs with about 87% of all cells having 0–5 PML-NBs and about 13% having 6–10 PML-NBs (Fig 1B and 1C). Cells treated with a non-invasive avirulent strain (BS176) showed a similar distribution of PML-NBs (Fig 1B and 1C). In contrast, M90T-infected cells had and overall 2-fold (*p* < 0.01) increase in the average number of PML bodies per cell (Fig 1B). This change is due to a significant (p<0.01) reduction in the percentage of cells with 1–5 PML-NBs and a significant increase in cells containing 11 or more PML NBs (p<0.01, Fig 1C). Since cells infected with BS176 had equal numbers of PML-NBs as uninfected cells, we suggest that post-invasion events of *Shigella* infection are required for numeric changes in these subnuclear domains. It is not clear why only a subset of M90T infected cells contain increased PML-NBs; however, since the BS176 and M90T strains also express RFP, we were able to confirm that all cells in the BS176 and M90T treated conditions (MOI = 10) were associated with these *Shigella* strains (S1 Fig). Further, the high PML-containing cells were associated with similar levels of M90T as normal PML-containing cells, suggesting that infection “load” *per se* does not correlate with PML-NB number. It is known that PML protein expression increases in response to cell cycle [40], interferon and other cellular stresses and signaling events [31], which in turn can result in increased PML-NBs. Alternatively, PML body number can increase without increasing PML protein levels through fission events in response to chromatin reorganization resulting from heat shock or DNA damage, resulting in the appearance of twin PML NBs or “doublets” [28, 41]. Alternatively, PML bodies may form through *de novo* nucleation from the soluble pool of PML, such as PML NB formation at telomeres [42]and association with herpes simplex virus type 1 (HSV-1) genomes [43]. To determine if the observed increase in PML NB number was due to increased PML expression or stability we analyzed control and infected cell lysates for PML levels (Fig 1D). We did not observe a significant change in overall PML protein levels or significant changes in PML isoform distribution during infection with either the non-invasive (BS176) or wild-type (M90T) *Shigella* strains. Furthermore, we observed multiple closely spaced PML-NB doublets in the infected cells as compared to controls, consistent with an increase in PML-NB number by body fission (Fig 1A, white arrows). Thus, these data indicate that the increase in PML body number occurs in the absence of increased PML protein expression, and may have occurred by PML NB fission or *de novo* PML body nucleation from soluble PML.

### *Shigella* infection alters SUMO1 localization and abundance

**Fig 2:**
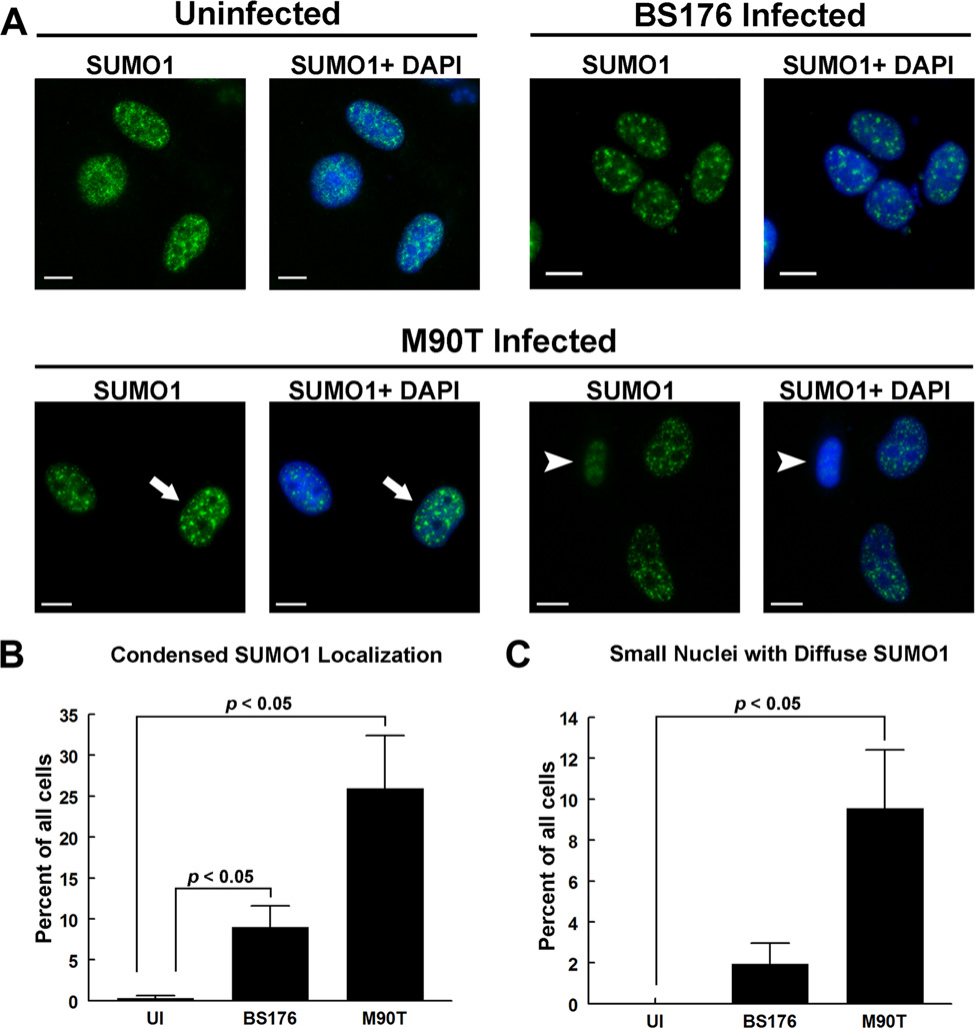
*Shigella* infection induces condensed localizations of SUMO1. HeLa cells were not infected (UI, uninfected), infected with a non-invasive strain (BS176), or infected with an invasive wild-type strain (M90T) as indicated, both strains contained a plasmid expressing the *afaE* adhesin (A). Cells were incubated for 3 hours, fixed and immunostained for SUMO1 (green). M90T-infected cells with condensed SUMO1 stain are indicated by arrows (left panels) while those with small nuclei and reduced SUMO1 staining are indicated with arrowheads (right panels). DNA was visualized with DAPI (blue). Scale bars = 10μm. The percentage of cells displaying either condensed SUMO1 staining (B) or small nuclei with reduced SUMO1 staining (C) for each treatment condition was quantified and the *p*-value of Student’s t-test was calculated. Values = mean +/- SE (n = 3).

In addition to investigating PML, we assessed *Shigella*-infected cells for changes in SUMO-1, an important post-translational modification involved in PML-NB function as well as important nuclear functions. We examined the localization of SUMO-1 in uninfected cells, cells infected with a non-invasive and non-virulent strain of *Shigella*, and a virulent and invasive strain of *Shigella*. In uninfected HeLa cells, SUMO-1 appeared largely in the nucleus and was present in diffuse foci (Fig 2A). This was also the case in cells infected with the non-virulent *S. flexneri* (Fig 2A, BS176). In contrast, in cells infected with wild-type *Shigella* SUMO-1 appeared as more condensed punctae with sharper edges and brighter staining (Fig 2A, M90T, left panels, arrows) in comparison to cells that were uninfected or infected with non-invasive *Shigella*. We quantified the percentage of cells displaying this SUMO-1 phenotype (Fig 2B) and noted a significant (*p* < 0.05) increase in condensed SUMO-1 in M90T-infected cells (~26% of cells) and BS176-infected cells (~9% of cells) when compared to uninfected cells in which less than 1% of cells showed the condensed SUMO-1 phenotype. SUMOylation of PML is important for the formation and stability of PML-NBs so we investigated if the increase in PML-NB might be due to an increase in the stabilizing effects of PML SUMOylation. We used immunofluoresence microscopy to determine if PML-NBs colocalized with sites of increased SUMO1 concentration in M90T-infected cells (S2 Fig). Although some SUMO1 could often be found associated with PML-NBs in uninfected and BS176-infected cells, PML was rarely present at areas of SUMO1 accumulation (S2A and S2B Fig). In M90T-infected cells with increased PML-NB number or with increased SUMO1 concentration, there was no obvious increase in PML association with SUMO1 structures or vice versa (S2C and S2D Fig). As well, our analysis did not reveal a correlation between PML body number and the condensed SUMO1 phenotype.

We also observed a second SUMO1 phenotype in M90T present in infected cells but not uninfected cells. Specifically, we observed some infected cells with nuclei that were smaller than the average nuclei in uninfected cells and that also showed reduced or absent SUMO1 signal (Fig 2A, M90T, right panels, arrowheads). We quantified the percentage of cells with this second SUMO1 phenotype (Fig 2C) and determined that ~9.5% of M90T-infected (*p* < 0,05 vs uninfected control) and ~2% of BS176-infected cells had small nuclei with reduced SUMO1 staining. This phenotype was not observed in any of the uninfected cells (Fig 2C). Furthermore, PML-NBs did not appear to either increase or decrease in these SUMO-depleted cells with small nuclei (S2E Fig).

**Fig 3:**
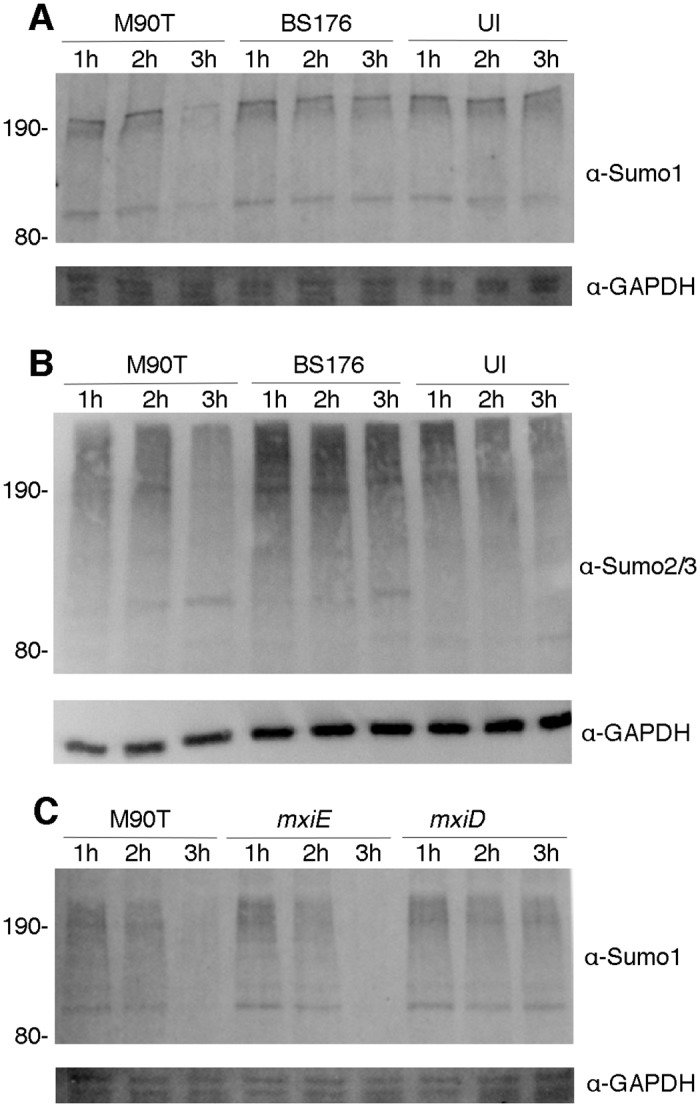
A decrease in SUMO-conjugated proteins accompanies *Shigella* infection. A comparison of SUMO-conjugated proteins from HeLa cells infected with wild type *Shigella* (M90T), a non-invasive strain (BS176), or uninfected cells for 1, 2, and 3 hours, both strains contained a plasmid expressing the *afaE* adhesin. Whole cell lysates were prepared from infected cells, separated on 7% polyacrylamide gels and immunoblotted using antibodies specific for SUMO1 (A) or SUMO2/3 (B). In (C) whole cell lysates from HeLa cells infected with wild type *Shigella* (M90T), a non-invasive mutant defective in the T3SS (*mxiD*), or an invasive mutant that does not produce a number of effectors (*mxiE*), was analyzed by immunoblotting using SUMO1-specific antibodies. Immunoblotting using GAPDH served as a loading control.

The SUMO1 localization phenotype associated with *Shigella* infection prompted us to investigate if, as with *L. monocytogenes*, *Shigella* infection resulted in a global impairment of SUMOylated proteins in infected cells. In HeLa cells infected for 3 hours, there was a marked reduction in the level of SUMO-conjugated proteins as evidenced by the loss of signal of large molecular weight proteins reacting with SUMO1–specific antisera (Fig 3A). This was also true for SUMO2/3 conjugated proteins, indicating that the general SUMO conjugating machinery may be affected (Fig 3B). To examine if *Shigella* factors that may be involved in the impaired SUMOylation, we infected Hela cells with a *mxiE* mutant and a *mxiD* mutant. The *mxiE* mutant is as invasive as wild-type *Shigella*, but does not express the full complement of *Shigella* effectors [44]. *mxiD* encodes an essential component of the T3SS apparatus and, as a result, *mxiD* mutants are non-invasive [45]. We observed that global SUMOylation was impaired in cells infected with the *mxiE* mutant (Fig 3C). In contrast, the *mxiD* mutant did not impair global SUMOylation (Fig 3C). We conclude that a functional T3SS is required for *Shigella* to impair SUMOylation.

**Fig 4:**
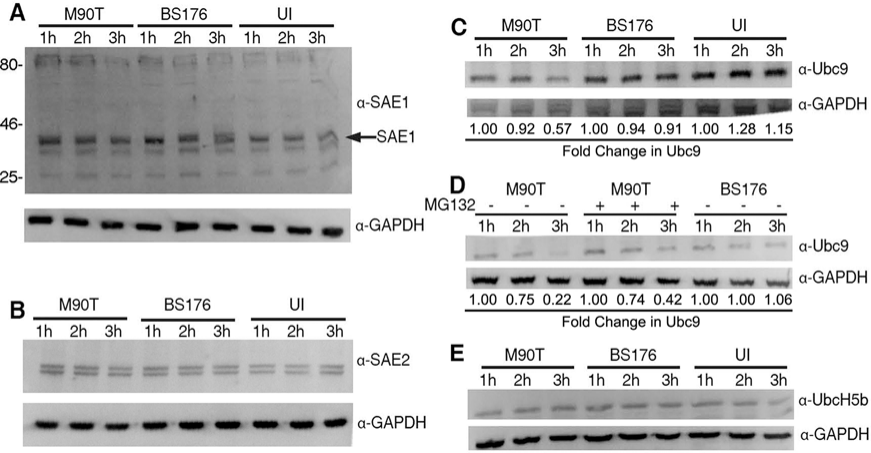
*Shigella* targets SUMO conjugating enzyme Ubc9. In (A-C) whole cell lysates prepared from HeLa cells infected for 1, 2 or 3 hours with the indicated strains of *Shigella* bearing a plasmid expressing the *afaE* adhesin were analyzed by immunoblotting using antibodies specific for SAE1 (A), SAE2 (B), or Ubc9 (C). In (D) HeLa cells were treated with the proteasome inhibitor MG132 prior to infection, or not, as indicated prior to infection and preparation of lysates. Lysates were analyzed for Ubc9 by immunoblotting. In (D) HeLa cells were infected with the indicated strains and lysates were analyzed by immunoblotting for UbcH5.

We examined the effect of *Shigella* infection on the stability of SUMOylation components. In cells infected with *Shigella*, the E1 activating enzymes SAE1 and SAE2 were readily detected in equal abundance up to 3 hours after infection (Fig 4A and 4B). In contrast, the SUMO conjugating enzyme Ubc9 decreased in cells infected with wild-type *Shigella* for 3 hours, while it remained constant in cells that had been infected with a plasmid-cured strain (Fig 4C). The decrease in Ubc9 was less pronounced in cells that had been infected with wild-type *Shigella* in the presence of the proteasome inhibitor MG132 (Fig 4D). We observed that the E3 ubiquitin conjugating enzyme UbcH5 was readily detected in all experimental conditions, indicating that Ubc9 is specifically destabilized (Fig 4E). We conclude that *Shigella* causes a decrease in global SUMOylation by destabilizing Ubc9.

Because the decrease in Ubc9 levels was inhibited by MG132, we hypothesized that Ubc9 was ubiquitinated in response to *Shigella* infection, thereby targeting it for destruction by the proteasome. We attempted to immunoprecipitate an epitope-tagged variant of Ubc9 to determine if it was ubiquitinated. However, we were unable to reliably detect Ubc9 from *Shigella*-infected cells. While our results indicate the depletion of Ubc9 protein levels in *Shigella*-infected cells is proteasome dependent, we have no evidence to support the modification of Ubc9 directly by either ubiquitin or the SUMO isoforms.

## Discussion

We demonstrate that *Shigella* infection impairs the SUMOylation pathway in infected cells. *Shigella* infection causes a redistribution of SUMO1 and the destabilization of the E2 conjugating enzyme Ubc9. *Shigella* infection also causes an increase in the amount of PML-NBs.

Although we cannot associate the observed PML and SUMO phenotypes with a benefit to either the host or the pathogen, this report is the first to demonstrate that bacterial pathogens can alter PML-NB number during infection. The increase in PML-NB number does not appear to be due to an increase in overall PML protein levels (Fig 1D). Therefore we speculate that *Shigella* infection may induce either a signaling and/or stress event (eg. cytokine signal or DNA damage) that alters PML-NB number, possibly through a fission event or *de novo* assembly of PML bodies from the soluble pool of PML protein. For example, we have demonstrated previously that PML-NBs respond to DNA damage by increasing in number, and *Shigella* is known to induce DNA damage signaling in infected cells [46]. In addition, it has been established that interferon signaling is required for restricting *Shigella* infection [47, 48]. Since PML is an interferon-regulated gene, it is also tempting to speculate that the observed PML-NB changes arise as a result of interferon signaling. Recently it was established that interferon acts through PML-NBs to enhance SUMOylation and inhibit viral replication [49]. We tried to correlate the *Shigella*-induced phenotypes of increased PML-NB number and decreased SUMO signal in cells but were unable to identify any clear trend to suggest that these phenotypes are connected.

Our results with the proteasome inhibitor MG132 suggest that Ubc9 is targeted by the proteasome, however we do not have direct evidence for ubiquitination of Ubc9. It is possible that MG132 inhibits a second protease, in addition to the proteasome, which is responsible for the destruction of Ubc9. It is also possible that a second protease cleaves Ubc9 initially, and the proteasome provides a mechanism for degrading the resulting peptide fragments, resulting in a method of degradation that is partially proteasome-dependent. Notably, the *Listeria*-dependent destruction of Ubc9 was shown to be insensitive to MG132 treatment [16]. There have been multiple reports of Ubc9 being targeted for destruction by invading pathogens [50]. Thus, it remains an open question as to whether Ubc9 is directly targeted by multiple pathogens, or if Ubc9 destabilization may be a general mechanism invoked by the host upon infection.

*Shigella* has efficient countermeasures against cellular programs such as autophagy and the NF-κB signaling that are involved in immune surveillance and bacterial clearance. Our results are in accord with the recently published findings demonstrating that SUMOylation restricts *Shigella* infection [38]. Inhibition of the SUMOylation pathway by *Shigella* requires a functional T3SS. This may reflect the need for *Shigella* to enter cells that may result in membrane damage or activation of intracellular immunity programs. Alternatively, *Shigella* may possess one or more effectors that are responsible for interfering with SUMOylation. The recent studies by Dunphy and coworkers show that successful pathogens can employ SUMOylation to regulate the fate of their virulence determinants [17]. This is akin to how bacterial pathogens manipulate the ubiqutin conjugation machinery is used to orchestrate effector function within infected cells [51, 52]. We note *Shigella* uses the effector OspF to effectively interfere with phosphorylation and the deamidase OspI to interfere with ubiquitination [8, 53]. Given that our data now indicates that *Shigella* targets another pervasive post-translational modification system (i.e. SUMOylation) of eukaryotic cells, it raises the possibility that this bacteria may have specific factors to manipulate the SUMOylation and/or the fate of SUMO-modified proteins to facilitate productive infection.

## Materials and Methods

### Strains and oligonucleotides

Streptomycin-resistant strain *Shigella* *flexneri* serotype 5a (M90T-Sm) was used [54] as a parent strain to create *mxiD::tetRA* mutants using lambda red recombination as described in [7]. Integration of the knock-out cassette at the desired location was confirmed by PCR using a primer common to the *tetRA* cassette and one upstream from *mxiD*. The *mxiE::kan* mutant was a gift from C. Parsot [44]. *Shigella* was routinely cultured in or on Trypticase Soy Broth (TSB) plus 0.01% Congo red, with or without 20 mg/mL agar. Tetracycline was used at a concentration of 5 μg/mL to select for the tetracycline resistance cassette (*tetRA*). Ampicillin, kanamycin and gentamicin were used at 100 μg/mL, 25 μg/mL and 15 μg/mL, respectively, to maintain plasmid selection when applicable.

#### Cell Culture and infections

Tissue culture cells were treated with 10 μM MG132, or not, one to two hours prior to infection. In all experiments, strains of *S. flexneri* bearing a plasmid encoding the gene *afaE* [55] were grown to mid-log phase and used to infected HeLa cells (obtained from ATCC CCL-2) at MOIs of 9.5. To ensure that *Shigella* entered HeLa cells we performed gentamycin protection assays in parallel to the samples that were used for analysis of proteins according to the methods described in Sidik[56]. We observed that when HeLa cells were treated with 10 μM MG132 for one hour prior to infection, *Shigella* entered with equal efficiency as those that were untreated. Strains used for immunofluorescence also encoded the gene dsRed on a plasmid [57]. Mammalian cells were incubated at room temperature for fifteen minutes prior to and after infection to synchronize invasion. Infected cells were then incubated at 37°C with 5% CO_2_ for between one and three hours, as indicated in the figures (Cells used for immunofluorescence were infected for three hours). Cells used for immunoblotting for PML were lysed in one microlitre of lysis buffer per thousand cells (50 mM Tris-Cl pH 6.8, 9M Urea), boiled for 10 minutes and sonicated. The protein concentration for the extracts were determined and equal amounts of protein were separated by SDS-PAGE. For immunoblotting for all other proteins, cells were lysed in 250 μL of SDS sample buffer (50 mM Tris-HCl pH 6.8, 2% SDS, 100 mM DTT, 10% glycerol), boiled for five minutes and separated by SDS-PAGE and blotted for specific antibodies and also GAPDH as a loading control.

#### Immunofluorescence

Protein localization was visualized by fluorescence microscopy. HeLa cells were plated onto coverslips in a 6-well plate and infected relevant bacterial strains. Three hours post-infection, cells were washed with PBS and fixed in 4% paraformaldehyde. Cells were permeabilized with 0.5% Triton X-100 in PBS, and blocked with 4% BSA in PBS. Cells were then immunolabeled with primary antibodies (1 h, diluted in blocking buffer), washed with PBS and incubated with fluorescently labeled secondary antibodies (45 min, diluted in blocking buffer). Following washes in PBS, cells were incubated with 1 μg/mL of 4',6-diamidino-2-phenylindole (DAPI) stain to visualize DNA. Fluorescent images were captured with Zeiss Axiovert 200M Microscope and Hamamatsu Orca R2 Camera or with Zeiss Cell Observer Microscope under a 63× immersion oil objective lens. Images were processed using only linear adjustments (e.g.brightness/contrast) with Slidebook (Intelligent Imaging Innovations, Boulder, CO) and Adobe Photoshop CS5. Quantification the SUMO1 phenotypes was conducted by coding the image filenames such that treatment condition parameters were concealed during analysis. At least 30 cells for each condition were assessed as either normal, having small nuclei with low SUMO1 expression or having condensed SUMO1. The samples were then decoded, organized by treatment category and the percentage of “low SUMO1” cells and “condensed SUMO1” cells were determined. Quantification of PML nuclear bodies was determined by counting the PML bodies in at least 30 cells per condition from each of three independent experiments. The average number of PML bodies was determined as was the percentage of cells containing either 0, 1–5, 6–10 or 11+ PML bodies. For PML and SUMO1 quantification, the means and standard errors from three independent experiments were calculated. Statistical significance was determined using the student's t-test in Microsoft Excel 2007.

#### Immunoblotting

Primary antibodies were purchased from the following sources: PML: Santa Cruz Biotechnologies product number sc-377390, Actin: Cell Signaling product number 4967, SUMO-1: Abcam product number ab32058, SUMO-2/3: Invitrogen product number 51–9100, SAE1: Abcam product number ab56957, SAE2: Imgenex product number IMG-5111A, Ubc9: Millipore product number MAB217, UbcH5b: Santa Cruz product number sc-100617, GAPDH: Ambion product number AM4300 and Abcam product number Ab9483. SDS-PAGE gels were transferred to PVDF membranes overnight using a wet-transfer apparatus, then blocked in 5% skim milk powder/TBST (145 mM NaCl, 5 mM Tris-Cl pH 7.5, 0.1% Tween-20, 5% skin milk powder) for one hour. Membranes were incubated in primary antibody for one hour, then washed four times for five minutes in TBST. Membranes were incubated in secondary antibody for one hour, then four additional five-minute washes were performed using TBST. Membranes were then developed using ECL Plus (Pierce) and imaged using a Kodak Electrophoresis Documentation and Analysis System or a Versadoc Imaging System (BioRad).

## Supporting Information

S1 Fig PML and SUMO1 effects are observed in *Shigella*-associated cells. Hela cells were not infected (A), infected with the non-invasive strain BS176 (B) or infected with the invasive wild-type strain M90T (C-E). Cells were incubated for 3 hours, fixed and immunostained for PML (red, alexa-649 labelled) or SUMO1 (green, alexa-488 labelled) as indicated. DNA was visualized with DAPI (blue) and RFP-expressing Shigella (BS176 and M90T) are shown in grayscale. Representative images of M90T-infected cells showing high PML (C), small nuclei with reduced SUMO1 (D) and condensed SUMO1 (E) are marked with an asterisk (*). Arrows indicate RFP-labelled bacteria associated with the asterisked cells. Scale bars = 10μm.
(TIF)

S2 Fig PML and SUMO1 show minimal colocalization. HeLa cells were not infected (A), infected with the non-invasive strain BS176 (B) or infected with the invasive wild-type strain M90T (C-E). Cells were incubated for 3 hours, fixed and immunostained for SUMO1 (green) and PML (red) as indicated. Representative images of M90T infected cells with the increased PML (C), condensed SUMO1 (D) and small nuclei with reduced SUMO1 (E) phenotypes are presented. DNA was visualized with DAPI (blue). Scale bars = 10μm.
(TIF)
