# Supplementary figures and images for: Shigella Infection Interferes with SUMOylation and Increases PML-NB Number
Source: PLoS One. 2015 Apr 7;10(4):e0122585. doi: 10.1371/journal.pone.0122585 (PMC4388590; doi:10.1371/journal.pone.0122585)

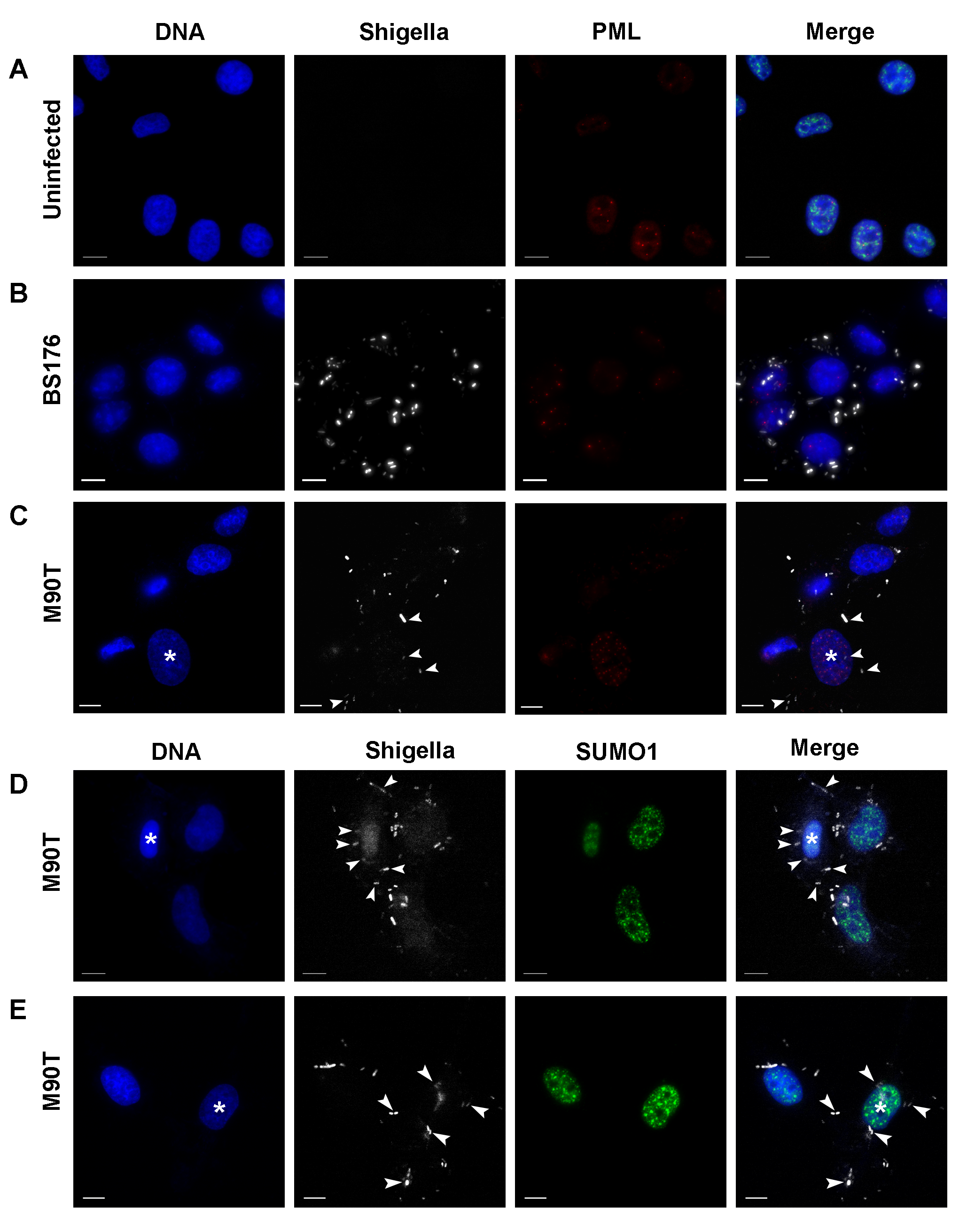

Supplement: S1 Fig — Hela cells were not infected (A), infected with the non-invasive strain BS176 (B) or infected with the invasive wild-type strain M90T (C-E). Cells were incubated for 3 hours, fixed and immunostained for PML (red, alexa-649 labelled) or SUMO1 (green, alexa-488 labelled) as indicated. DNA was visualized with DAPI (blue) and RFP-expressing Shigella (BS176 and M90T) are shown in grayscale. Representative images of M90T-infected cells showing high PML (C), small nuclei with reduced SUMO1 (D) and condensed SUMO1 (E) are marked with an asterisk (*). Arrows indicate RFP-labelled bacteria associated with the asterisked cells. Scale bars = 10μm. (TIF) [file pone.0122585.s001.tif]

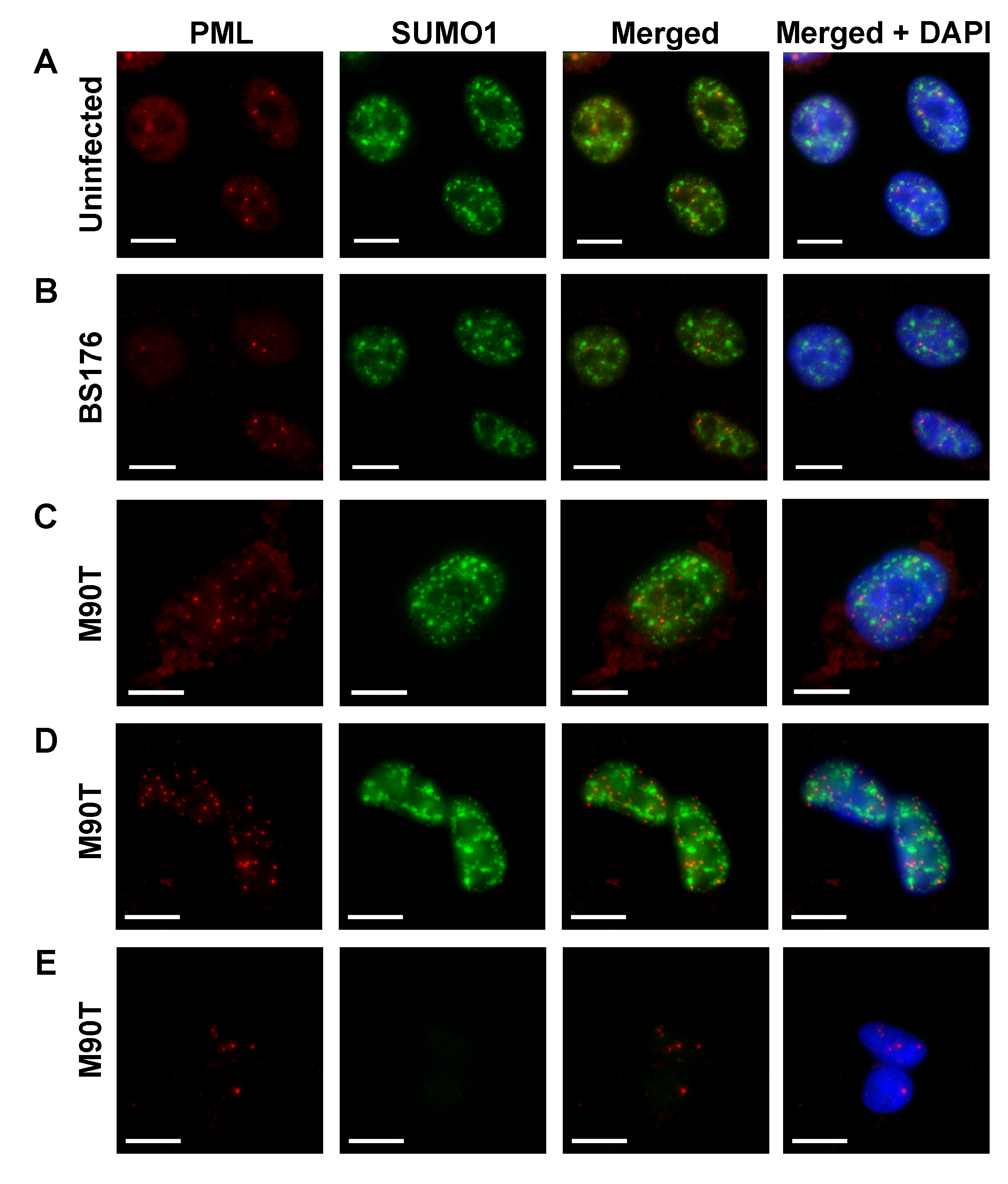

Supplement: S2 Fig — HeLa cells were not infected (A), infected with the non-invasive strain BS176 (B) or infected with the invasive wild-type strain M90T (C-E). Cells were incubated for 3 hours, fixed and immunostained for SUMO1 (green) and PML (red) as indicated. Representative images of M90T infected cells with the increased PML (C), condensed SUMO1 (D) and small nuclei with reduced SUMO1 (E) phenotypes are presented. DNA was visualized with DAPI (blue). Scale bars = 10μm. (TIF) [file pone.0122585.s002.tif]
